# Why does caloric restriction increase life and healthspan? The ‘clean cupboards’ hypothesis

**DOI:** 10.1093/nsr/nwaa078

**Published:** 2020-04-29

**Authors:** John R Speakman

**Affiliations:** State Key Laboratory of Molecular Developmental Biology, Institute of Genetics and Developmental Biology, Chinese Academy of Sciences, China; Institute of Biological and Environmental Sciences, University of Aberdeen, UK; CAS Center of Excellence in Animal Evolution and Genetics, Kunming Institute of Zoology, Chinese Academy of Sciences, China

## Abstract

The disposable soma hypothesis explanation of the effects of caloric restriction (CR) on lifespan fails to explain why CR generates negative impacts alongside the positive effects and does not work in all species. I propose here a novel idea called the clean cupboards hypothesis which overcomes these problems.

## BACKGROUND

The effects of caloric restriction (CR) on longevity were discovered 100 years ago [1]. Since then the effect has been replicated in a wide variety of animals (reviewed in [2]). More recent work in non-human primates provides a complex picture but also indicates that there are some beneficial impacts on both healthspan and lifespan [3–6]. Impacts on human lifespan will likely never be systematically studied, but a short-term randomised controlled trial produced indicative changes in important biomarkers of ageing [7,8]. There are also suggestions that naturally restricted human populations show increased longevity [9].

The question of why CR has these effects can be answered at several different levels. For example, there is a debate over whether the effect is due to a deficit in calories or protein [10,11]. There is also the issue of the exact molecular mechanism. Finally, we may consider the evolutionary function of the effect: why did the responses to CR evolve? In this perspective I will address this latter issue and propose a novel hypothesis for why CR affects lifespan. I have called the hypothesis the ‘clean cupboards hypothesis’ for reasons that will become apparent later. This is an important question to answer because our understanding of why CR influences life and healthspan has ramifications for whether we expect CR to have similar effects in humans. Since, as noted above, a direct study of the impact of CR on human lifespan is unlikely to ever be conducted, we are reliant on this theoretical understanding of the function of CR to make inferences about whether it is worth pursuing as a human lifespan intervention, although if it leads to other benefits it may be worth pursuing anyway.

**Figure 1.**
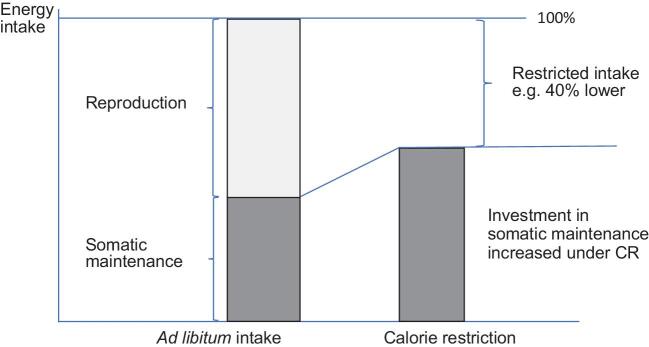
Schematic diagram of the disposable soma interpretation of the effects of CR. Under *ad libitum* feeding energy is allocated to both reproduction (light grey) and somatic maintenance (dark grey). When the total supply is reduced under caloric restriction the animal switches off reproduction but increases allocation to somatic maintenance. This causes the extended health and lifespan effects. For a similar conceptual diagram see Adler and Bonduriansky (2014) [25].

The main evolutionary hypothesis for the effect of CR on lifespan is derived from the disposable soma hypothesis (DSH) [12–14]. The DSH is a classical trade-off model that posits energy resources are limited and hence animals must make an evolutionary decision about how to use them. There are two main uses: somatic maintenance and reproduction. If an animal invests in somatic maintenance it improves survival probability, but it does so at the cost of reproduction. Alternatively, investing heavily in reproduction can only occur at the costs of somatic maintenance and hence survival. The DSH therefore explains the phylogenetic inverse correlation between reproductive output and lifespan [15]. This theory also provides a potential evolutionary explanation of what is happening during CR. It is presumed that wild animals would only experience CR as a temporary phenomenon. Since attempts to reproduce under such limited energy supply would likely fail, animals are better served by switching off reproductive investment completely and diverting all their resources into somatic maintenance. This would maximise their chances of surviving the period of restricted energy supply. This resource allocation model is illustrated in Fig. 1. In the laboratory the lean period never ends, and the animals keep the somatic maintenance activities switched on indefinitely, leading to the lifespan increase [14]. This idea is consistent with direct observations that exposure to CR reduces reproductive investment [16], and potentially explains why CR may be less effective in males, which expend less energy on reproduction ([17] but see review [11] suggesting no sex difference in the response).

This DSH interpretation of the evolutionary background to the function of CR leads to the prediction that because the costs of reproduction in small animals are substantially higher (relative to baseline non-reproductive costs) than the reproductive costs in larger animals, the savings that can be made by switching off reproduction and diverting energy to somatic maintenance are considerably larger in smaller animals. Hence it is argued we observe a large effect of CR in small mammals like mice [18], rats [19] and very small primates [6], but in larger animals the impact is attenuated [3] or lost altogether [4] (but note the interpretation of these primate studies is complicated [5]). Thus in humans where investment in reproduction is also relatively small [20] we would anticipate a small or zero impact. That is, the impact of CR on lifespan and healthspan is likely to depend critically on the background life history of the species involved [14,21–24].

In this paper I question this interpretation of the evolutionary background to the CR effect. Several previous authors have also questioned it, by suggesting CR is an artefact of captive housing [25], where animals are not exposed to patterns of mortality typical in the wild. That is, because animals in captivity do not die of predation and disease, it is possible for the later life benefits of restriction—on e.g. cancer susceptibility—to emerge, but in the wild it would never happen. The suggestion is that the response to restriction does not reflect the levels of nutrients per se, but the consequent changes in signals such as the mTOR and Insulin signalling pathways that may mediate the lifespan effect. However, while this interpretation may be correct it does not answer the central issue of the evolutionary background to why nutrient depletion activates such systems.

## SOME PROBLEMS WITH THE DISPOSABLE SOMA INTERPRETATION OF THE IMPACT OF CR

**Figure 2.**
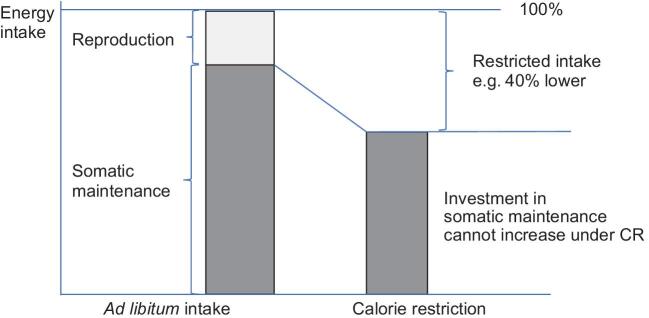
The reality of energy investment in small mammals experimentally exposed to caloric restriction. Normally animals in CR protocols are restricted from reproducing when in the *ad libitum* state. Hence the investment in reproduction is small. Consequently, even if this investment is switched off there is no scope in the budget to increase allocation to somatic maintenance.

Probably the biggest problem with the DSH interpretation of the effects of CR (Fig. 1) is that it may apply to how animals respond to restriction of food intake in the wild, but this is radically different from how CR is applied in the laboratory (particularly in studies of mammals), which is where the lifespan and healthspan effects of CR are observed. This is because in CR experiments the *ad libitum* condition refers to animals that are prevented from reproduction. While there may be some energy being invested to maintain the reproductive organs, sustain spermatogenesis in males etc., the cost of reproduction is much reduced (Fig. 2). It is extremely unlikely that in such animals that are prevented from reproducing that investment in reproduction is greater than 10% of total energy use. Therefore, even if this reproductive investment is completely switched off, if the total energy coming in under restriction is reduced by more than 10% there is no scope to increase the investment in somatic maintenance. Moreover, under the model in Fig. 1, as the level of restriction increases the amount of energy that can be allocated to somatic maintenance gets progressively lower. The prediction then is that the benefits of restriction should also get lower as the level of restriction increases. In fact exactly the opposite is the case: the greater the level of restriction the longer the animals live [11,26,27]. The DSH interpretation is therefore at odds with several fundamental characteristics of the phenomenon.

## A NEW IDEA: THE CLEAN CUPBOARDS HYPOTHESIS

**Figure 3.**
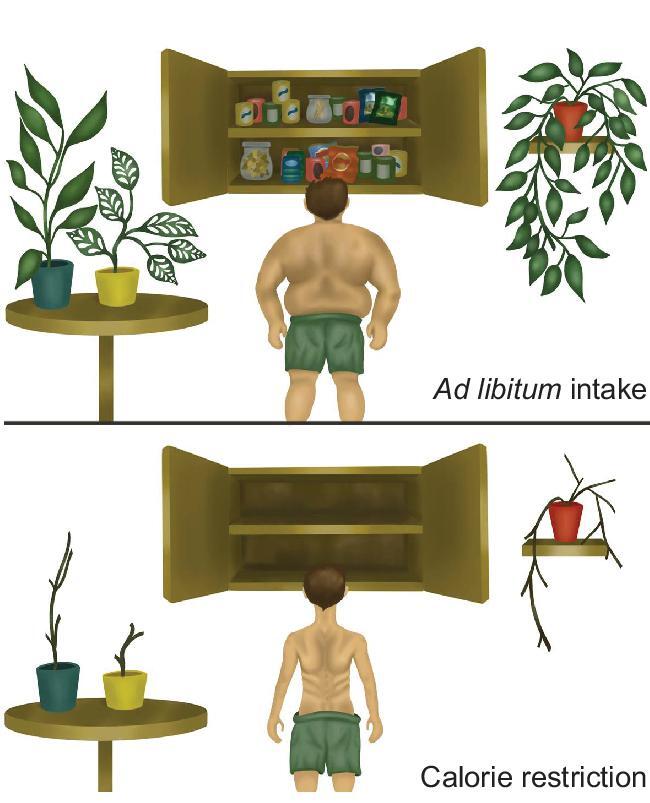
The ‘clean cupboards’ hypothesis for the effects of calorie restriction suggests that under restriction individuals act only to try and make an energy balance to survive the immediate crisis. The graphical abstract illustrates an analogous situation of a person locked in their home with insufficient food to eat. In response to the period of caloric restriction they will eat all the stored food in cupboards in their house. So incidentally under restriction they get a set of spectacularly clean cupboards compared to when they were eating *ad libitum*. They didn't set out with the aim of cleaning their cupboards but it was a positive epiphenomenon of trying to make an energy balance. They may also end up eating all their house plants as well. That would be a negative outcome. The model predicts CR will lead to a mix of positive outcomes (clean cupboards) and negative outcomes (dead plants). Artwork by Stephanie Summers.

Imagine that you are under house arrest. It is okay though because every day someone comes to your house and gives you the food you need for that day. The food matches exactly your energy requirements. Suddenly one day the person brings the food minus one of the components. Now you have a problem because the food supply does not meet your requirements. To make up the shortfall you might draw down your fat reserves a little, but if this happened several days in a row then you would start to get hungry. If you are anything like me, however, there is lots of food in your house stashed away in the cupboards of your kitchen, fridge, pantry, etc. So what you would do is make up the shortfall partly by using your fat stores, but also by eating some of that food. If the shortfall continued what you would do is continue to make it up by eating into this hoard. After a couple of months passes by you will have completely cleaned out all the kitchen cupboards and eaten every little last bit of food in the fridge and pantry. At this stage you might get a bit desperate and start to eat your house plants to satisfy the hunger (Fig. 3). An incidental by-product then of you having to make an energy balance each day is that you have generated a set of spectacularly clean cupboards (but no house plants). You did not set out with this aim. There was no long-term strategy involved. All you wanted to do was stop getting hungry each day. As soon as the supply of food resumes you will start to pack away any excess food back into these stores, and buy some new plants. Eventually the cupboards will be just as full and messy as they were before.

I suggest here that this is a partial analogy for what animals are doing under CR. That is, when we place them under CR they have an immediate issue—they need to make an energy balance. So what they do is eat into their reserves. Like you they have a reserve of stored body fat, and they draw on this [28]. But the animal can also derive energy by clearing out the junk that has built up in the tissues and cells throughout their bodies. This includes things like misfolded proteins and damaged organelles which can provide energy through upregulation of autophagy, and senescent cells that can also be dismantled. This is consistent with the fact that both autophagy and removal of senescent cells are both upregulated under CR [29–32]. The important point is that the animal has no long-term strategic aim in doing this. All it is doing is trying to make an immediate energy balance. However, a completely coincidental side effect of cleaning out the rubbish is that the animal lives longer. The lifespan and healthspan benefits of CR are like the clean cupboards. We did not aim to get them, they were an emergent property of trying to make an energy balance each day. This contrasts with the interpretation from the DSH that autophagy and removal of senescent cells are a deliberate strategy to extend lifespan by protecting the soma. Moreover, another consequence of the food shortage in the clean cupboards analogy was you not only cleaned the cupboards, but you also ate all the house plants. This emphasises that CR is predicted by this model not only to generate a range of positive outcomes, but also some negative effects. These are not predicted by the DSH.

To clarify, by saying the lifespan and healthspan benefits of CR are like ‘clean cupboards’, this does not mean to imply that they are equivalent and exchangeable. I am only saying that they are both happy positive coincidences of trying to make an energy balance. Doing one set of things to generate an energy balance may have lifespan consequences, while doing other things for the same reason may have healthspan benefits. The mechanism underlying healthspan and lifespan effects does not have to be the same. It is like eating food stored in the pantry and eating food in the fridge. They both contribute to the energy shortfall but they have different consequences—one cleans out the fridge and the other cleans out the pantry.

The ‘clean cupboards’ analogy is only a partial analogy because in animals under CR the process of reducing fat and lean tissues reduces energy requirements. Calculations suggest this remodelling of tissue is enough to bring energy demands down sufficiently to match the lowered food supply [33]. At this point the animal is back into a steady state where its incoming food matches its energy demands. This re-establishment of a steady state explains why the level of garbage clearance matches the level of restriction and hence there ends up being a positive relationship between the level of restriction and the lifespan enhancement. If this is correct then the benefits of CR will be independent of the level of reproductive investment, and hence CR should be as beneficial to humans as it is to small rodents.

The essential difference between these two ideas is that the DSH posits increased investment in some component of the energy budget that increases lifespan. The clean cupboards idea on the other hand suggests animals only do things under restriction to make a positive contribution to their energy imbalance. This new hypothesis can be refuted then if it was to be shown there is some process switched on under restriction that requires *increased* energy investment but results in improved lifespan. We know for example that under restriction the alimentary tract enlarges [27]. This investment however seems designed to extract more energy from the lowered food supply [27] and hence has a net energy benefit. It seems elevated clearance of senescent cells and upregulated autophagy probably come under the same banner—but quantitative data on this are currently lacking. Similarly, increased physical activity under restriction [34] may involve energy investment, but this is likely done to try and find additional food to ingest.

Another idea explaining the effects of CR on life and healthspan is hormesis [35,36]. This differs from the model being proposed here in several respects. Under the hormesis interpretation CR creates a stressor that the animal responds to by switching on protection and repair mechanisms. This then leads the animal to be more resilient to other stressors, by the hormesis effect, and this leads to the health and lifespan benefits. This differs in two key respects from the clean cupboards idea. First, the animal is investing in protection and repair with a view to enhancing its survival. Second, the knock on effects are all postulated to be positive. In contrast, the clean cupboards idea posits animals under CR only invest in things that have a net energy release, and in doing this there may be a range of positive and negative outcomes (like the missing houseplants).

Uniquely then this idea explains why caloric restriction results in a number of negative outcomes that are incompatible with the idea that the animals are increasing their investment in survival (Fig. 1). These include retardation of wound healing [37] and the ability to fight off infections [38, 39]. By the ‘clean cupboards hypothesis’ these are problems that arise because the animal is switching off costly processes like sustaining immune functions to save energy. In the clean cupboards analogy these are the eaten house plants—the undesirable side effects.
